# Stabilization of 2D Raft Structures of Au Nanoclusters with up to 60 Atoms by a Carbon Support

**DOI:** 10.1002/smsc.202400093

**Published:** 2024-05-22

**Authors:** Sean Lethbridge, Theodoros Pavloudis, James McCormack, Thomas Slater, Joseph Kioseoglou, Richard E. Palmer

**Affiliations:** ^1^ Nanomaterials Lab Mechanical Engineering Swansea University Bay Campus Fabian Way Swansea SA1 8EN UK; ^2^ Department of Physics Aristotle University of Thessaloniki GR‐54124 Thessaloniki Greece; ^3^ Advanced Imaging of Materials Swansea University Bay Campus Fabian Way Swansea SA1 8EN UK; ^4^ Cardiff Catalysis Institute School of Chemistry Cardiff University Cardiff CF10 3AT UK; ^5^ Center for Interdisciplinary Research and Innovation Aristotle University of Thessaloniki GR‐57001 Thessaloniki Greece

**Keywords:** 2D rafts, AC‐STEM, DFT calculations, nano‐clusters

## Abstract

Herein, the stabilization of 2D single‐atom high gold rafts containing up to ≈60 Au atoms on amorphous carbon, fabricated by sputtering of atoms and imaged by aberration‐corrected scanning transmission electron microscopy, is demonstrated. These rafts deviate from the established cluster transition from 2D to 3D Au structural motifs in free clusters, which occurs in the region of 10–14 atoms. The experimental findings are supported by explicit ab initio calculations of Au_*n*_ (*n* = 3–147) clusters on graphene and the role of cluster–surface interactions in the stabilization of the 2D single‐atom high Au cluster rafts on graphene is revealed. The transition from equilibrium 2D–3D structures is delayed to *n* = 19, while metastable 2D single‐atom high rafts compete with 3D structures up to about *n* = 60 atoms. The catalytic activity of supported nanoclusters depends strongly on their structure (and carbon‐based supports are used for a number of reactions); therefore these results are relevant to the catalytic performance of nanocluster‐based catalysts.

## Graphical abstract

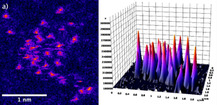

## 1 Introduction

The structure of nanoclusters determines their physical and chemical properties across numerous electronic, magnetic, optical, catalytic, and sensing applications.^[^ ^1^ ^]^ Specifically for catalysis, the catalyst's structure is immensely important, since it determines the number of surface sites available for catalysis and gives rise to distinct surface structures that control the activity and selectivity of a nanoparticle (NP) catalyst.^[^ ^2^, ^3^ ^]^ For example, 2D bilayers of gold atoms have been shown to possess a higher catalytic activity for carbon monoxide oxidation than near‐spherical clusters or single atoms.^[^ ^4^ ^]^ Therefore, the determination of the most stable structures of nanoclusters (in particular 2D versus 3D) is of high importance in determining catalytic performance. Au NPs display catalytic activity for a number of reactions, not just the oxidation of CO. A number of reasons have been proposed; but the number of low‐coordinated Au atoms in nanostructures is proposed as the main factor.^[^ ^5^ ^]^

The equilibrium structure of Au nanoclusters has been extensively investigated both theoretically and experimentally over the past two decades. Here, we provide a detailed summary as a solid base for the new results we report. In refs. [6, 7], the evolution of structural motifs of free Au_*n*_ clusters (*n* = 11–24 and *n* = 15–24, respectively) was studied through a comparison of experimental measurements (electron diffraction and photoelectron spectroscopy) with density‐functional theory (DFT) calculations. These studies reported a transformation from 2D to 3D structures in the range *n* = 12–14, the development of cage structures for *n* = 16 and 17, the appearance of a tetrahedral structure at *n* = 20, and the emergence of a highly symmetric tubular structure for *n* = 24. Johansson et al.^[^ ^8^ ^]^ using a genetic algorithm (GA) coupled with DFT calculations, found that 2D and 3D structures are almost isoenergetic for neutral Au_11_, while larger clusters are 3D. Kinaci et al. employed DFT calculations taking into account van der Waals (vdW) interactions and a modified GA to investigate the structures of Au_12_, Au_13_, and Au_14_. They found that globular and planar structures coexist; the transition from 2D to 3D happens gradually.^[^ ^9^ ^]^ In ref. [10], Nhat et al. found the geometric and energetic properties of Au_*n*_ clusters (*n* = 2–20) via DFT calculations and reported 2D geometries up to Au_11_, the transition from an oblate form to a pyramid at Au_17_ and tetrahedral evolution for Au_17_–Au_20_ by adding atoms to the Frank–Kasper 16‐vertex. Yen et al.^[^ ^11^ ^]^ combined density‐functional tight‐binding and DFT calculations with a novel optimization algorithm and predicted the transition of Au clusters from a planar (*n* = 3–11) to oblate‐like cage (*n* = 12–15), then hollow cage (*n* = 16–18), and finally a pyramidal‐like cage (*n* = 19, 20). Similarly, Wu et al. found a heavy bias of DFT predictions toward 2D structures of Au clusters and circumvented it by incorporating nonlocal effects in their simulations.^[^ ^12^ ^]^ They predicted a 2D to 3D transition at Au^12−^ and Au^8+^ as well as Au^10^ for neutral clusters. In ref. [13], Goldsmith et al. predicted the structures of neutral gas phase Au_*n*_ clusters (*n* = 5–13) via *ab initio* molecular dynamics simulations which take into account vdW interactions and a comparison with experiments. They reported a change from 2D to 3D structures at Au_11_ when the temperature was 100 K and the coexistence of 2D and 3D structures for Au_8_, Au_9_, and Au_10_ at 300 K. Xu et al. predicted planar structures for both ground and excited states of Au_*n*_ from *n* = 2 to 13 clusters using a combination of an artificial bee colony algorithm and first‐principles calculations.^[^ ^14^ ^]^ In ref. [15], Deng et al. using DFT calculations coupled with a revised particle swarm optimization algorithm, confirmed the generally reported transition from planar to spherical shape for Au cluster size between 11 and 14 atoms. Rare exceptions to the consensus are refs. [16, 17], where Nhat et al. via coupled‐cluster theory (CCSD(T)) and DFT calculations, predicted 3D structure for Au_10_ and Au_12_ clusters.

From the references discussed, it is clear that the majority of theoretical work seems to agree on the cluster size of the 2D to 3D transition in free gold clusters (10–14 atoms). However, experimental work shows the existence of planar Au clusters at larger sizes than this. In ref. [18], Wang and Palmer report dynamic fluctuations of the Au_20_ cluster structure (on amorphous‐carbon transmission electron microscopy (TEM) grids), in which tetrahedral projections appear only in ≈5% of frames, and the majority of images show disordered structures, including planar ones. Similarly, in ref. [19], a fluctuating dynamic behavior of size‐selected Au_55_ clusters is demonstrated via aberration‐corrected scanning transmission electron microscopy (ac‐STEM). Chiral structures and similar sister isomers are identified for the Au_55_ clusters, while a significant number of other amorphous‐like and 2D structures are recorded. Importantly, almost no high‐symmetry structures (face‐centered cubic [fcc], icosahedron, or decahedron) are observed. Again clusters were deposited on an amorphous carbon support in both microscopy studies. Theoretical studies have so far failed to predict the emergence of 2D structures at larger sizes (above 14 atoms); therefore, we hypothesize that the role of the support is critical in the structural evolution of Au clusters.

Synergistic interactions between the nanoclusters and the support may alter the physical properties of the clusters.^[^ ^20^ ^]^ Interaction with the support can affect the clusters’ structural properties, especially at small sizes, stabilizing metastable structures and playing a crucial role in the morphology of the nanostructured system. An example of a special cluster‐support interaction is that of Au clusters deposited on graphene: the triangular lattice of the (111) surface of the fcc metals is a good symmetry match for the hexagonal honeycomb lattice of graphene, and Au is accommodated on graphene with small lateral strain, relatively weak binding and large Au–C equilibrium separation.^[^ ^21^, ^22^, ^23^ ^]^ An example of how the support changes the transition from 2D to 3D structures on other supports is given in ref. [24], where Au_*n*_ (*n* = 1–20) on rutile TiO_2_ (110) surfaces was found to prefer planar or quasi‐planar structures.

In this work, we report an abundance of 2D Au clusters grown on amorphous carbon up to ≈Au_60_ identified via high‐angle annular dark field (HAADF) imaging in ac‐STEM. We find 2D clusters persisting throughout the size regime considered, alongside 3D structures. Large‐scale *ab initio* calculations quantify from first principles the structural evolution of Au clusters from 2D to 3D structures both in free‐space and on a graphene support, which is used instead of amorphous carbon in DFT calculations due to the complexity of employing an amorphous carbon model (but demonstrating the effect of a carbon‐based support). We examine trigonal, hexagonal, pyramidal, cage, tetrahedral, octahedral, decahedral, and icosahedral clusters with 3–147 atoms and establish the effect of the model graphene support on the cluster size at which a transition from 2D to 3D clusters is observed.

## 2 Results and Discussion

**Figure 1:**
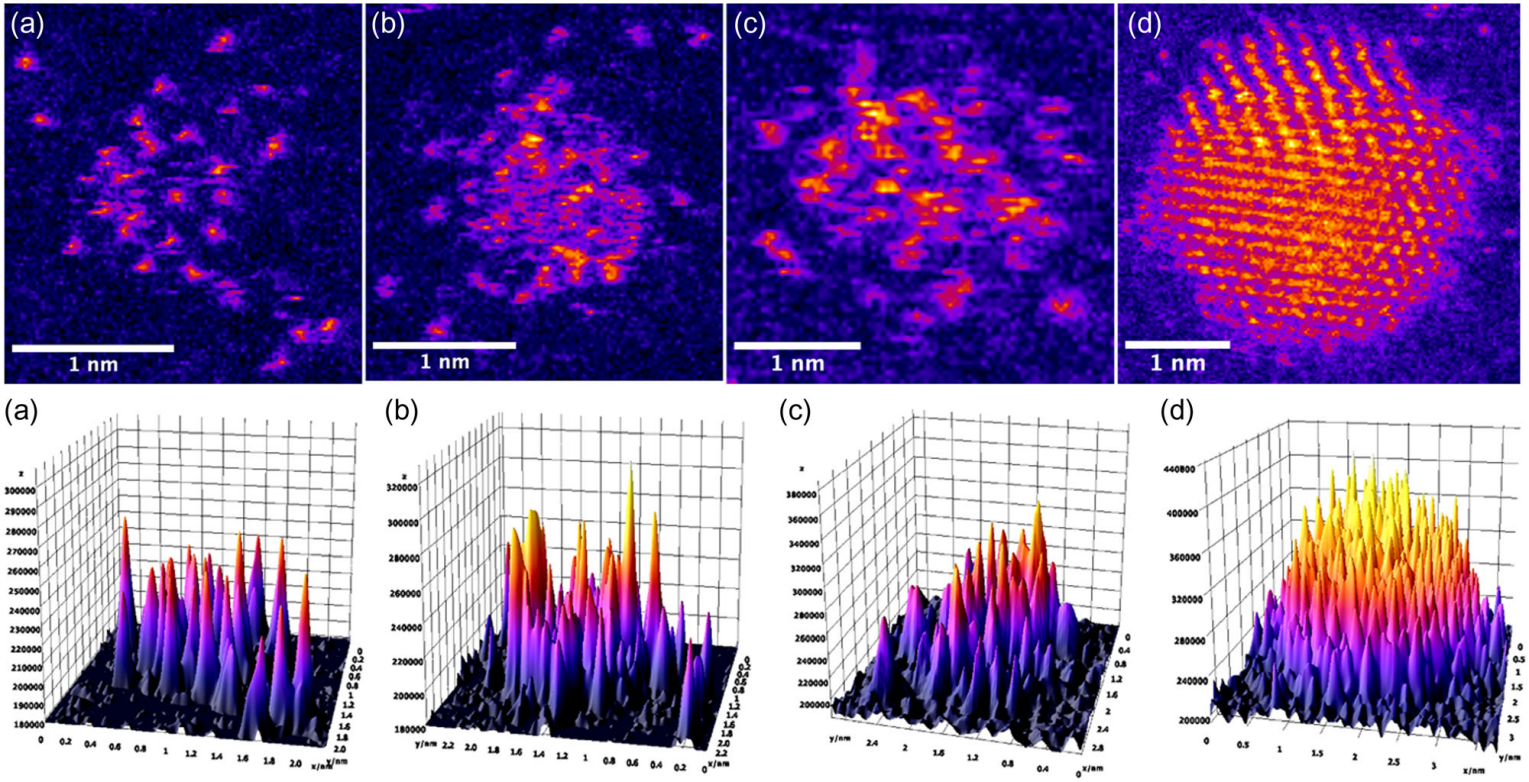
HAADF–STEM images and 3D surface plots of samples made by sputtering Au atoms onto an amorphous carbon support film. The sample contains isolated single atoms, a–c) 2D “rafts”, and d) 3D clusters. The figures shown demonstrate 2D and 3D structures of sizes a) Au_16_, b) Au_54_, c) Au_83_, and d) Au_1322_.

Aberration‐corrected HAADF–STEM images of clusters formed from individual atoms sputtered onto amorphous carbon reveal a mixture of single atoms, 2D “rafts” of atoms and clearly three‐dimensional clusters (**Figure** **1**). The 2D rafts can be identified as such because their individual pixel intensities are no greater than that of single atoms. Analysis across HAADF–STEM images gives an approximate peak pixel intensity value for 2D rafts corresponding to that of a single‐Au atom when the average background carbon support intensity is subtracted. This is in line with the intensity difference between the Au rafts and carbon support, confirming the approximately monolayer thickness of the raft structures. These 2D rafts can further be realized when viewed as 3D surface plots. Modeling in this way better aids the differentiation of 2D from 3D structures, thus improving identification.

**Figure 2:**
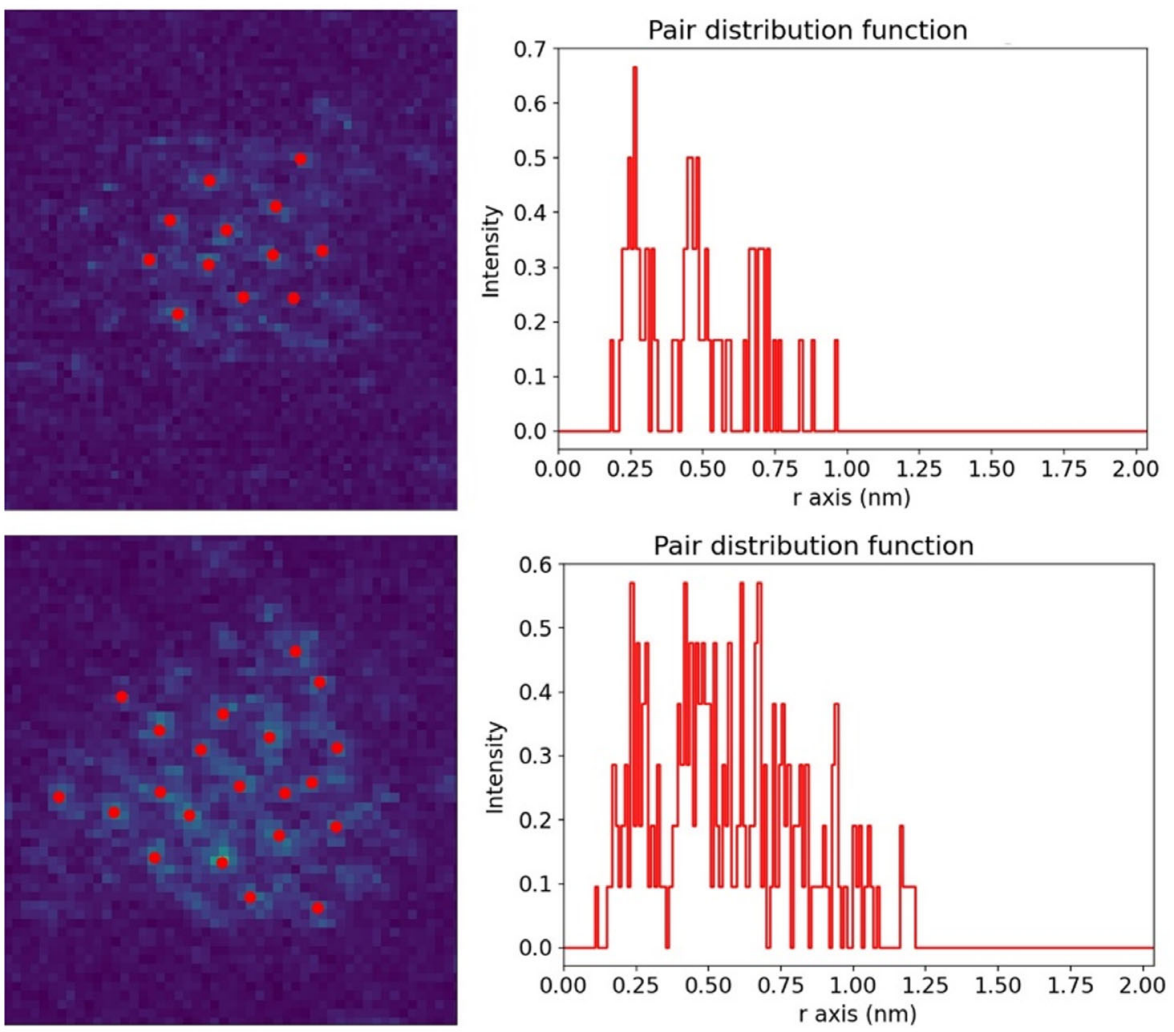
Pair distribution function analysis of two “raft‐like” structures, demonstrating a clear peak in atomic spacing at 2.5 Å.

The pair distribution functions of two of the 2D rafts were obtained from HAADF–STEM images (**Figure** **2**) and show a clear peak at ≈2.5 Å, which is close to the nearest neighbor spacing of fcc Au at 2.884 Å. The reduction in nearest neighbor distance can be accounted for by the cluster–surface interaction with the underlying carbon film; the unit cell size is 2.46 Å for graphite. This supports the suggestion that the 2D raft structures are stabilized via lattice matching with the carbon substrate. The clear peak in the pair distribution function at this spacing is indicative that atoms are closely packed within the same 2D plane. We found that all 2D rafts are nominally “glassy” in terms of long range structure, i.e., they do not possess an extended crystalline structure. But even in 3D Au clusters, amorphous structures are the most common structural motif found for sizes below 309 atoms,^[^ ^18^, ^19^ ^]^ as predicted theoretically for size 13 clusters.^[^ ^25^ ^]^ No transition between 2D and 3D structures was observed under the electron beam during imaging. Motion of atoms at the edges of 2D and 3D structures was observed, with sputtering of single atoms away from the structures sometimes seen when imaging over multiple frames. Images were acquired without prolonged exposure to the electron beam to minimize such sputtering.

**Figure 3:**
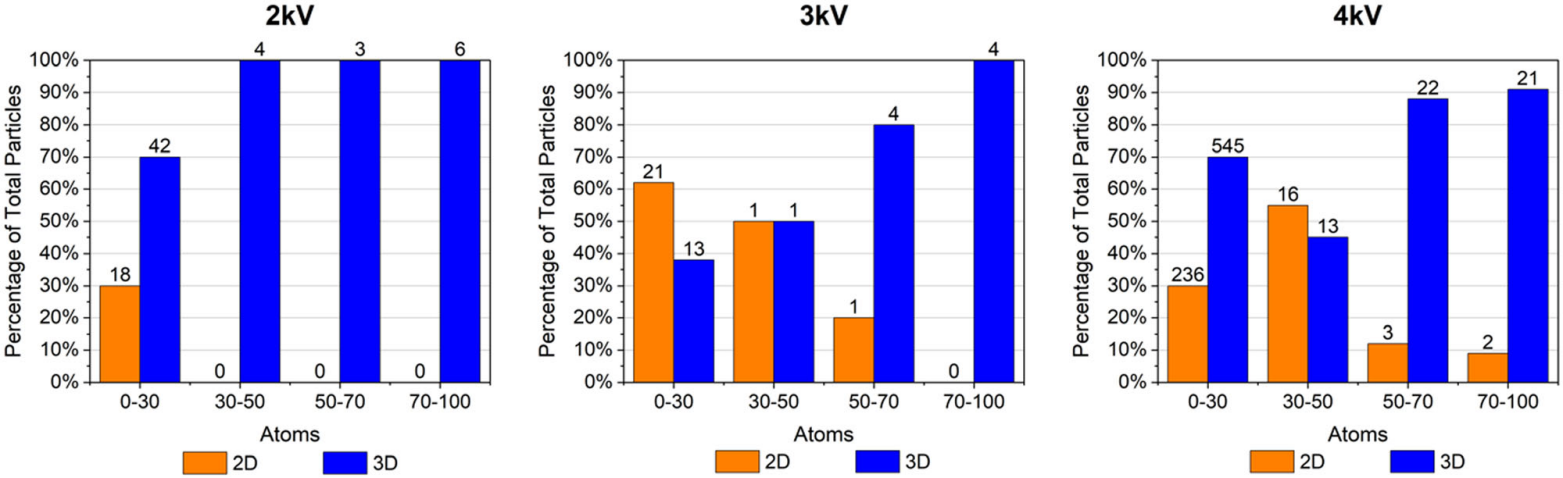
Plots of 2D (orange columns) versus 3D (blue columns) cluster structure populations for three different sputtering voltages. The 2D rafts are found at up to ≈83 atoms in size at 4 kV sputtering voltage, but only at smaller sizes at 2 and 3 kV.

To quantify the number of atoms in each raft or 3D cluster, we calibrated the intensity of the features in the HAADF images using the average integrated intensity of over 100 single atoms. From this calibration, we could obtain the number of atoms in each raft or cluster to characterize the typical size at which there is a transition from 2D to 3D structure. The number of atoms in 2D rafts could be confirmed by counting the number of atoms observable within the raft and matching this to the number found using the calibrated intensity. Classification of clusters up to 100 atoms in size into either 2D or 3D structures revealed the approximate size at which 2D structures were no longer found (**Figure** **3**). A high percentage of these clusters are identified as 2D, particularly for higher sputtering voltages and smaller cluster sizes. The former is probably due to the fact that a higher sputtering voltage in this case equates to a high sputtering rate, which means atoms land on surfaces in quick succession and are more likely to form metastable structures, hence 2D structures are emphasized. Larger 2D rafts may also be abundant at 4 kV due to the much larger number of particles imaged. In all cases, no clusters larger than 86 atoms are identified as 2D, with the largest 2D raft being 30 atoms at 2 kV, 54 atoms at 3 kV, and 83 atoms at 4 kV.

**Figure 4:**
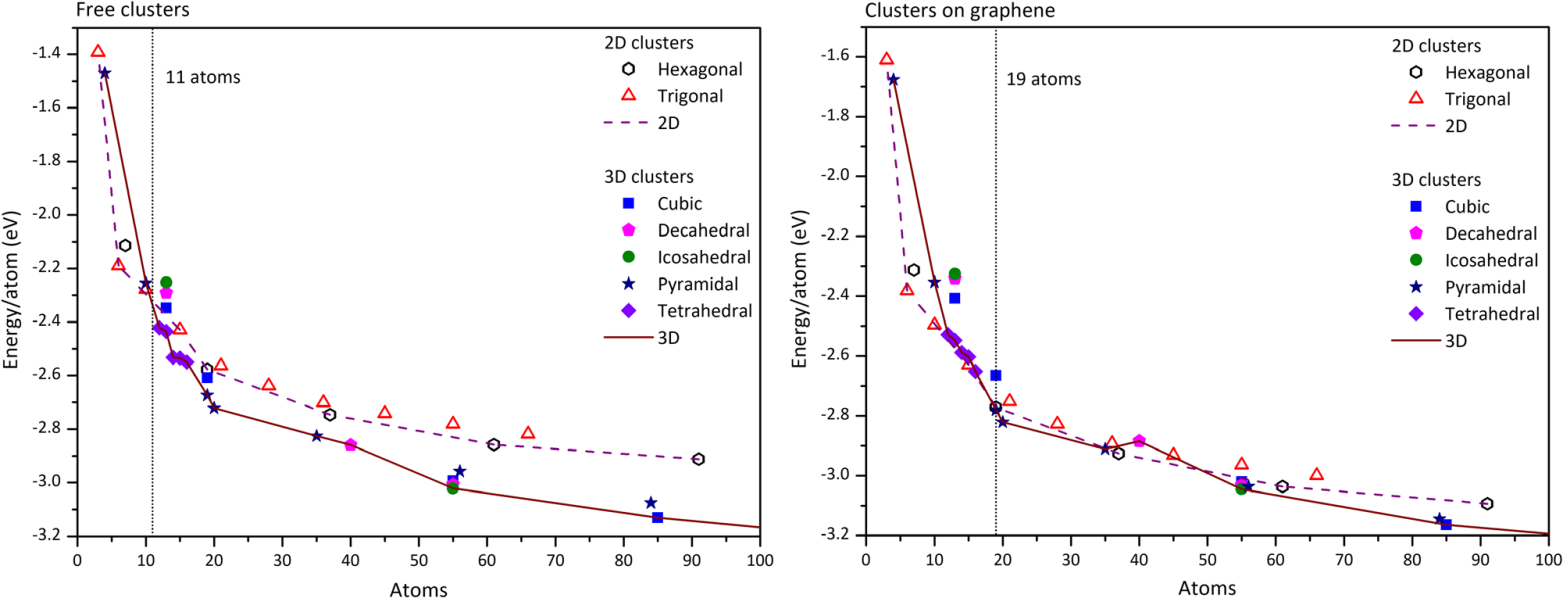
The energy per atom of free (left) and supported on graphene (right) Au_*n*_ clusters (*n* = 3–100), calculated by DFT. The transition from 2D to 3D structures happens at ≈11 atoms in the left chart but at ≈19 atoms in the right chart. The 2D and 3D motifs remain highly competitive up to 60 atoms when the clusters are supported on graphene.

In **Figure** **4**, the energy per atom calculated by DFT for all the clusters examined in the model calculations is shown. Small 3D clusters prefer the cage, tetrahedral, and pyramidal motifs, while larger 3D clusters prefer the highly ordered octahedral, decahedral, and icosahedral motifs and their truncations. The trigonal and hexagonal 2D structures are always very close energetically, both for free clusters and clusters on graphene. The transition in free clusters from these 2D structures to 3D structures happens very early, at ≈11 atoms, as previously reported in the literature (and summarized earlier). However, when the same clusters are placed on graphene, this 2D–3D transition is delayed and happens at ≈19 atoms. This implies that even the weak interaction between Au and graphene is enough to stabilize the 2D clusters. Interestingly, even for sizes above the transition, the various 2D and 3D motifs remain very similar in energy when the clusters are on graphene. The energy difference between them is within ≈0.08 eV. A hexagonal 2D cluster of 37 atoms is even preferable compared with the 3D pyramidal and decahedral clusters at 35 and 40 atoms, respectively. This behavior persists up to ≈60 atoms. After this point, the energy difference increases, and the 3D structures become clearly preferable energetically in the calculations. Interestingly, this is a lower value than results found experimentally (2D structure of 83 atoms found). This could be attributed to the limitations of modeling on graphene, where an amorphous carbon‐based model could possibly demonstrate a further delayed transition from 2D to 3D structures.

One point of interest is that the clusters in the experimental images are not of a precisely symmetric shape. However, many of them exhibit a quasi‐hexagonal shape, which is in agreement with the calculations to the extent that the hexagonal 2D rafts are of generally lower energy compared with their trigonal counterparts.

**Figure 5:**
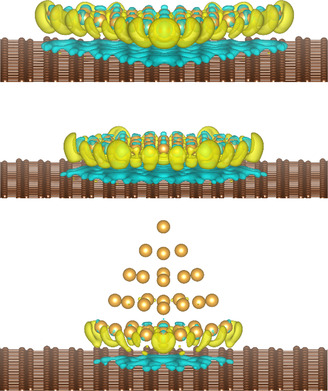
The charge density difference of a trigonal (top), a hexagonal (middle), and a pyramidal (bottom) Au cluster on graphene. The clusters comprise 36, 37, and 35 atoms, respectively. Blue and yellow colors represent charge depletion and accumulation, respectively.

The relative stabilization of 2D versus 3D gold cluster shapes on the carbon surface in the calculations is related to the number of atoms participating in the Au_*n*_–graphene bond. In **Figure** **5**, an illustration of the charge transfer (an illustration of the bonds formed) between three 2D and 3D clusters of similar sizes and the graphene sheet is shown. When the clusters are deposited on graphene, a charge transfer from the graphene to the cluster is observed. For example, each Au atom of the hexagonal Au_37_ cluster gains ≈0.1 *e*. Additionally, the charge distribution between the Au atoms changes slightly. For example, the charge is distributed more or less evenly in the free Au_37_ cluster, with the central atoms being ≈0.01 *e* richer than the peripheral atoms. The same difference for the deposited cluster is ≈0.05 *e*. Virtually all the atoms of a 2D cluster interact with the carbon atoms beneath them. However, in 3D clusters, only the bottom layer of atoms is in contact. For the clusters shown in Figure 5 by way of example, this translates to all the atoms of the Au_36_ trigonal and Au_37_ hexagonal clusters, but only 15 atoms for the Au_35_ pyramidal cluster. More carbon dangling bonds are saturated by the 2D gold clusters; therefore, the 2D cluster energies decrease by a larger margin on graphene. For 3D clusters on graphene, their energy is typically ≈0.10–0.05 eV lower than their free counterparts depending on their size, while for 2D clusters the corresponding value is ≈0.20 eV. However, as the clusters grow further in size, this energy reduction due to the passivation of the dangling bonds is overcome by the energy reduction due to the steadily increasing number of Au–Au bonds in the 3D clusters.

In summary, 2D Au rafts are observed experimentally up to ≈50–90 atoms in size on the amorphous carbon surface, alongside 3D cluster structures. The maximum size of the observed 2D structures is similar to the size at which 3D structures are clearly energetically preferred to 2D structures in our model calculations of Au clusters on graphene. The transition size predicted by calculations of free clusters is considerably smaller and emphasizes the need to include the cluster–surface interaction in calculations of supported clusters.

## 3 Conclusions

We have observed 2D rafts of Au with sizes approaching 100 atoms in structures grown from individual Au atoms sputtered onto an amorphous carbon film. The persistence of 2D nanocluster structures to this size is at odds with previous calculations of free cluster energetics, and our own. For free clusters, a clear transition from 2D to 3D structures occurs in the region of 10–14 atoms. To explore the effect of the carbon surface at a qualitative level, we have reported calculations of the Au cluster energies on a model graphene surface where the support stabilizes 2D raft structures compared with 3D structures up to the higher size of 19 atoms. In future, it would be fascinating to calculate the catalytic activity of these rafts compared with their 3D counterparts, in terms of both turnover and selectivity. In general, we may expect a notable effect. Corresponding experimental studies will require the preparation of “structurally‐pure” cluster arrays, or perhaps single NP experiments. Ab initio DFT studies of a support as complex as amorphous carbon itself may come into view as computing power continues to increase.

## 4 Experimental Section

4.1

4.1.1

##### Theoretical Details

The DFT calculations were performed using the Vienna *Ab initio* Simulation Package (VASP)^[^ ^26^, ^27^ ^]^ under the Perdew–Burke–Ernzerhof derivation of the Generalized Gradient Approximation (GGA‐PBE),^[^ ^28^ ^]^ with Projector‐Augmented‐Wave (PAW) pseudopotentials.^[^ ^29^, ^30^ ^]^ The energy cutoff of the plane‐wave basis set was 400 eV. The Brillouin zone was sampled at the Γ point and the break condition for the electronic self‐consistent loop was 1 meV. The width of the vacuum surrounding the nanoclusters was more than 15 Å in all directions, which ensured that there was no interaction between the nanoclusters and their nearest image. Van der Waals interactions were taken into account through the DFT‐D3 method^[^ ^31^ ^]^ with Becke–Johnson damping.^[^ ^32^ ^]^ Under these settings, the lattice constant of the Au unit cell was found to be 4.101 Å and the *a* lattice constant of the graphene unit cell was found to be 2.468 Å.

The choice of graphene as a model substrate was made due to size limitations—amorphous carbon itself would not be feasible. A realistic representation of such a substrate would require several disordered carbon layers and multiple amorphous configurations, which would add hundreds, if not thousands, of atoms to the calculation. The calculations performed in this work were already large scale: even the one graphene layer used comprised 704 atoms. However, we expected the results to be relevant because a‐C contained graphitic regions. Moreover, the cluster footprint in this work was small so the area beneath each cluster might be locally quite flat, in essence quite similar to graphene, and thus individual atomic potentials were similar. We believe that this compromise comparison still allowed us to understand the effect of the carbon support, without precisely matching structures or energies, and thus demonstrated how the effect of graphitic patches could cause 2D Au raft structures to grow. We built models of trigonal (3, 6, 10, 15, 21, 28, 36, 45, 55, and 66 atoms), hexagonal (7, 19, 37, 61, and 91 atoms), octahedral (13, 19, 55, 85, and 147 atoms), decahedral (13, 40, 55, 127, and 147 atoms), icosahedral (13, 55, 135, and 147 atoms), and pyramidal (4, 9, 10, 19, 20, 35, 56, and 84 atoms) clusters, plus the pyramidal and tetrahedral structures reported in ref. [6] (12, 13, 14, 15, and 16 atoms). Since the highly ordered structures (octahedral, decahedral, and icosahedral) were known to be stable for small and large clusters and the 3D structures from ref. [6] were the best of a “large number” of candidates, we believed that the 42 model clusters (15 2D and 27 3D) were sufficient to reveal the general behavior of small Au clusters. Images of the atomistic models are included in the article's Supporting Information.

The trigonal and hexagonal 2D NPs were interfaced to graphene in accordance with the energetically favorable configuration of refs. [23, 24]: the atoms of these (111) slabs were accommodated alternately on top of C atoms and in the middle of C hexagons. This particular configuration forced a lattice constant of 4.031 Å on the gold lattice—down from 4.101 Å. The initial strain was then only ≈2% for the perfect pairing of the two surfaces. The equilibrium distance between the NPs and the graphene sheet was ≈3.30 Å, the same value previously reported in the literature.^[^ ^23^, ^24^ ^]^ The octahedral, decahedral, and icosahedral NPs were interfaced with one of their (111) facets on graphene in the same way. After placing the clusters on graphene, the models were relaxed with all the atoms in the simulations allowed to move. In agreement with previous works on large Au_561_ clusters on graphene,^[^ ^26^ ^]^ we observed no wetting in these small clusters.

The cluster energies $E_{\text{cluster}}$ were calculated according to

(1) $$E_{\text{cluster}}=E_{\text{cluster}+\text{graphene}}-E_{\text{graphene}}$$

where $E_{\text{graphene}}$ and $E_{\text{cluster}+\text{graphene}}$ are the total energies after the relaxations for the pristine graphene sheet and the cluster–graphene combination, respectively.

The charge density difference Δρ was calculated according to

(2) $$\Delta \rho =\rho_{\text{cluster}+\text{graphene}}-\rho_{\text{cluster}}-\rho_{\text{graphene}}$$

where $\rho_{\text{cluster}+\text{graphene}}$, $\rho_{\text{cluster}}$, and $\rho_{\text{graphene}}$ are the electron charge densities obtained from static runs of the relaxed models of the NP–surface combination, the free NP and the free surface, respectively. The imaging of the results was performed with the VESTA visualization program.^[^ ^33^ ^]^

##### Experimental Details

The sputtered atom source produces an Ar^+^ beam using a cold cathode ion gun (Scienta Omicron ISE 5) typically operated at 5 keV. The ion beam strikes the gold target (99.999% pure gold) at 45°, with a beam diameter at the target of 10–15 mm, producing a secondary flux of gold atoms onto the holey amorphous TEM carbon substrate. The gold atoms were deposited at thermal energies, thus ensuring surface deposition, not implantation, in which previous work has demonstrated the pinning energy of Au to be ≈18.6 eV atom^−1^.^[^ ^34^, ^35^, ^36^ ^]^ The argon ions were accelerated at 2, 3, 4, and 5 kV with a flux of 10–20 μA each to bombard the gold target. The sputter currents were measured at the target using an Agilent 34401 A multimeter. The source was operated in high vacuum with base pressure around 5 × 10^−9^ mbar and argon pressure under operation around 5 × 10^−5^ mbar at room temperature. Subsequently, the sputtered atoms form gold clusters on the surface of a holy carbon film (on a copper TEM grid). The sample was exposed to the beam for 15 min. Enough material was sputtered to observe a wide range of cluster sizes, from single atoms to clusters consisting of >1000 atoms.

HAADF images were acquired on a probe‐corrected JEOL Grand ARM STEM operated at 300 kV at the electron Physical Sciences Imaging Centre facility at Diamond Light Source. A convergence semi‐angle of 26 mrad, a beam current of 25 pA, and a detector inner semi‐angle of 58 mrad were used for all the data collection. The level, gain, and dwell time of all images were kept constant to allow quantitative comparison of intensities between images.

Quantification of numbers of atoms in an image region was performed using a similar approach to that used previously in ref. [37], where the calibration was performed using existing size‐selected clusters. The approach used here, however, was based on single‐Au atoms; a background‐subtracted intensity of each cluster was calibrated using an average intensity of a set of identifiable single atoms (also background‐subtracted). To ensure accuracy in single‐atom intensity calibration, this process was repeated for all the identifiable single atoms in each image, where atoms were identified as high intensity regions with total areas between 0.01 and 0.1 nm^2^. The order of a few hundred atoms was identified per image. The final intensity values were plotted on a histogram, where the position of the first Gaussian peak was assigned as the average intensity value of single atoms. The calibrated single‐atom intensity was then used to quantify the number of atoms in rafts and clusters in the image, given that image intensity of HAADF imaging scaled linearly with number of atoms in clusters up to size of at least 1000 atoms.^[^ ^38^ ^]^

To confirm that the spacing between atoms was consistent with locations in the same plane for 2D rafts, we conducted a 2D pair distribution function analysis of two of the raft structures we observed. This analysis was carried out by manually marking the position of atoms using the atomap python package, before using the pair_distribution_function function also contained in atomap.^[^ ^39^ ^]^

## Conflict of Interest

The authors declare no conflict of interest.

## Author Contributions

T.P., T.J.A.S. and R.E.P.: Conceptualization; T.P. and J.K.: methodology; T.P. and J.K.: software; T.P., S.L., J.M.C. and T.J.A.S.: validation; T.P., S.L., J.M.C. and T.J.A.S.: formal analysis; T.P., S.L., J.M.C. and T.J.A.S.: investigation; T.J.A.S., J.K. and R.E.P.: resources; T.P. and S.L.: data curation; T.P., S.L. and T.J.A.S.: writing—original draft preparation; T.P., S.L., T.J.A.S., J.K. and R.E.P.: writing—review and editing; T.P. and S.L.: visualization; R.E.P.: supervision; R.E.P.: project administration; T.P., T.J.A.S. and R.E.P.: funding acquisition. All authors have read and agreed to the published version of the manuscript.

## Supporting information

Supplementary Material

## Data Availability

The data that support the findings of this study are available from the corresponding author upon reasonable request.
