# Supplementary material for: Stabilization of 2D Raft Structures of Au Nanoclusters with up to 60 Atoms by a Carbon Support
Source: Small Sci. 2024 May 22;4(8):2400093. doi: 10.1002/smsc.202400093 (PMC11935196; doi:10.1002/smsc.202400093)
Supplement: Supplementary file 1 — Supplementary Material [file SMSC-4-2400093-s001.pdf]

Supplementary Information

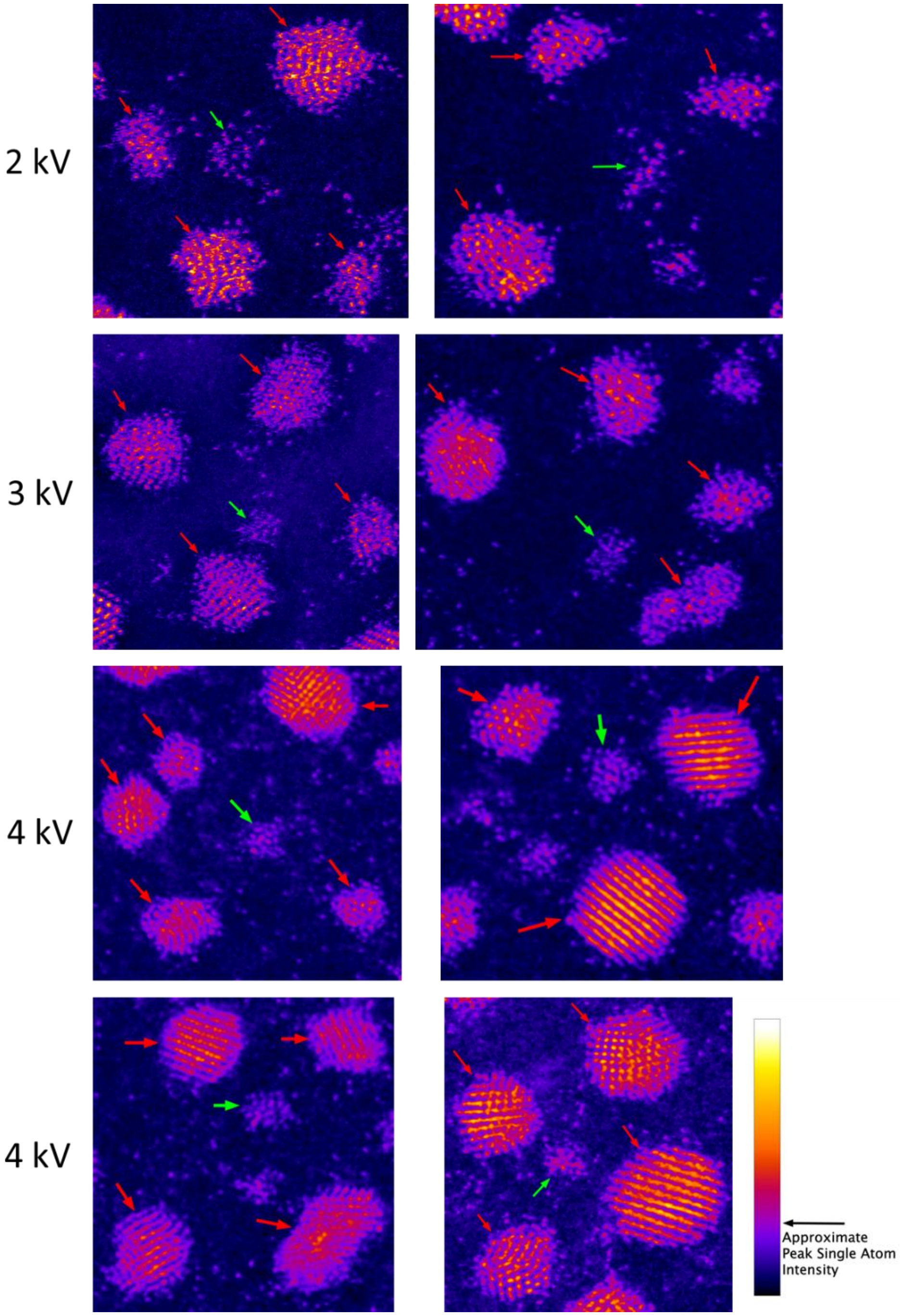

Figure S1. HAADF-STEM images of a sample made by sputtering Au atoms onto an amorphous carbon support film for three different sputtering voltages (2, 3 and 4 kV). The samples contain isolated single atoms, two-dimensional “rafts” (green arrows), and three-dimensional clusters (red arrows). The HAADF intensity is represented by the colour bar shown on the right, in which an approximate maximum single Au atom intensity is identified.

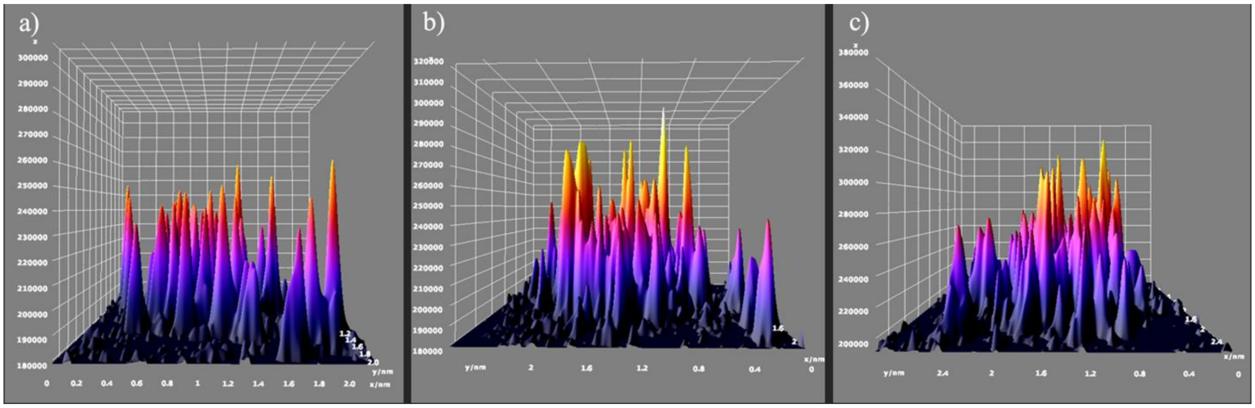

Figure S2. Side view surface plot images of 2D Monolayer Rafts presented in Figure 1 demonstrating a peak intensity difference corresponding to that of a single atom.

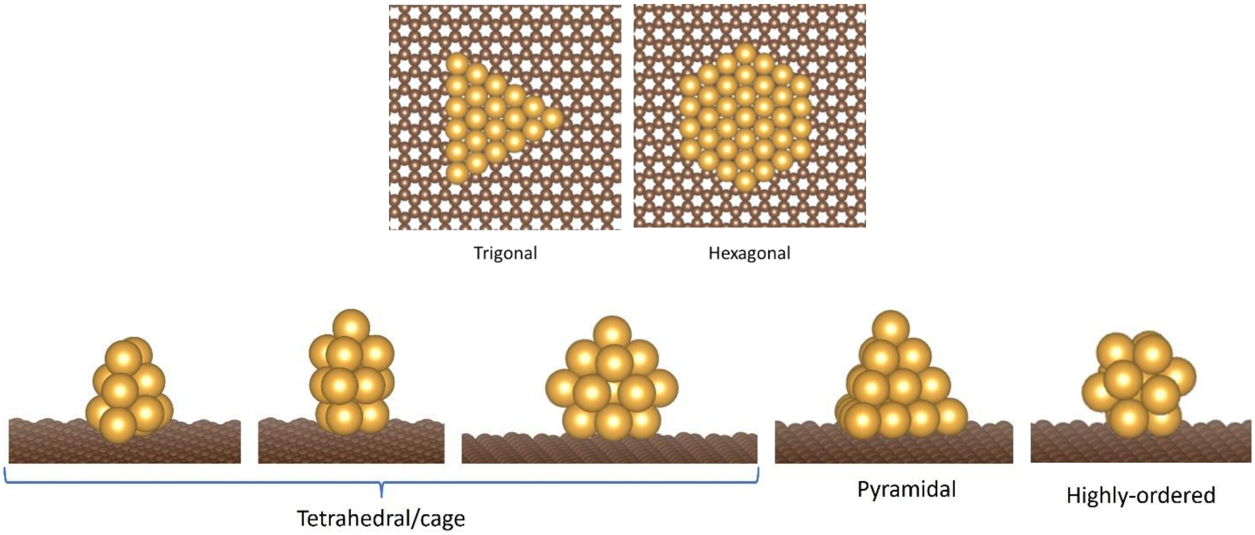

Figure S3. Images of the families of the 2D and 3D DFT models after relaxation. Highly-ordered structures may be of octahedral, decahedral, or icosahedral shape and their truncations.
